# The mutational spectrum of hunter syndrome reveals correlation between biochemical and clinical profiles in Tunisian patients

**DOI:** 10.1186/s12881-020-01051-9

**Published:** 2020-05-24

**Authors:** L Chkioua, O Grissa, N Leban, M Gribaa, H Boudabous, H Ben Turkia, S Ferchichi, N Tebib, S Laradi

**Affiliations:** 1 Research Laboratory of Human Genome and Multifactorial Diseases, Faculty of Pharmacy, University of Monastir, Street Avicenne, 5000 Monastir, Tunisie; 2 Department of Cytogenetic and Reproductive Biology Farhat HACHED Hospital, Sousse, Tunisia; 3 Laboratory of pediatrics, La Rabta Hospital Tunis, Tunis, Tunisia; 4 Biochemistry Laboratory, Farhat HACHED Hospital Sousse, Sousse, Tunisia; 5 The Auvergne-Rhône-Alpes Regional Branch of the French National Blood System EFS/GIMAP-EA 3064, 42100 Saint Etienne, France

**Keywords:** Mucopolysaccharidosis type II, Hunter syndrome, Clinical features, Mutations

## Abstract

**Background:**

Mucopolysaccharidosis type II (MPS II) or Hunter syndrome is an X-linked recessive lysosomal storage disorder resulting from deficient activity of iduronate 2-sulfatase (IDS) and the progressive lysosomal accumulation of sulfated glycosaminoglycans (GAGs).

**Methods:**

A diagnosis of MPS II or Hunter syndrome was performed based on the following approach after a clinical and paraclinical suspicion. Two biochemical and molecular tests were carried out separately and according to the availability of the biological material.

**Results:**

All patients in this cohort presented the most common MPS II clinical features. Electrophoresis of GAGs on a cellulose acetate plate in the presence of a high concentration of heparane sulfate showed an abnormal dermatan sulfate band in the patients compared with that in a control case. Furthermore, leukocyte IDS activity ranged from 0.00 to 0.75 nmol/h/mg of leukocyte protein in patients.

Five previously reported mutations were identified in this study patients: one splice site mutation, c.240 + 1G > A; two missense mutations, p.R88P and p.G94D; a large deletion of exon 1 to exon 7; and one nonsense mutation, p.Q396*. In addition, two novel alterations were identified in the MPS II patients: one frame shift mutation, p.D450Nfs*95 and one nonsense mutation, p.Q204*.

Additionally, five known *IDS* polymorphisms were identified in the patients: c.419–16 delT, c.641C > T (p.T214M), c.438 C > T (p.T146T), c.709-87G > A, and c.1006 + 38 T > C.

**Conclusions:**

The high level of urine GAGs and the deficiency of iduronate 2-sulfatase activity was associated with the phenotype expression of Hunter syndrome. Molecular testing was useful for the patients’ phenotypic classification and the detection of carriers.

## Background

Hunter syndrome (MPS II; OMIM 309900) is an X-linked recessive inborn error that causes deficient activity of iduronate 2-sulfatase (IDS, EC3.1.6.13). This lysosomal enzyme hydrolyses the 2 sulfate groups of the L-iduronate 2-sulfate units, dermatan sulfate and heparan sulfate [1] .

The *IDS* gene, located on chromosome Xq28, contains 9 exons and is transcribed into a 1400-bp mRNA, which encodes a precursor protein (UniProt: P22304) of 550 amino acids [2].

More than 640 different mutations (www.hgmd.org, 2017) in the *IDS* gene have been reported in patients with Hunter syndrome, including small deletions, altered splicing, gross deletions, small insertions, complex rearrangements, small indels, and gross insertions/duplications [3]. MPS II presents both severe and mild clinical subtypes [1]. The severe phenotype of MPS II, the neuropathic form, is characterized by a progressive clinical deterioration with neurological involvement, multiple dysostosis including joint stiffness, coarse facies including broad noses, macroglossia, and cardiovascular involvement that often leads to death before 15 years of age. Diagnosis is often completed at 3 years of age. Patients with the mild phenotype of MPS II have minimal or no neurological deterioration; they are characterized by joint stiffness and relatively mild somatic changes. In the most attenuated form of MPS II, diagnosis may not be made until 10 years of age, and death may occur in early adulthood; however, some patients have survived until their fifth or sixth decades of life [4].

Based on clinical manifestations of MPS II patients, the biochemical analyses i.e., quantitative and qualitative urinary glycosaminoglycan (GAG) concentration, are usually performed first. This preliminary screening requires a differential diagnosis with the Hurler syndrome (MPS I) for which we obtained the same GAG profile. Thus, the measurement of IDS enzyme activity is necessary to confirm the diagnosis. The genetic test of the *IDS* gene is important for prenatal diagnosis in MPS II families.

This study aims to describe the mutational spectrum of the Hunter syndrome to elaborate the possible correlation between biochemical and clinical profiles in Tunisian patients.

## Methods

### MPS II patients

This is a series of cases of patients recruited at a young age (1–2 years) in the paediatric departments of various hospitals in Tunisia: Tunis, Sousse, Sfax and Kairouan.”

Most of the MPS II patients were from consanguineous marriages. There was no known relationship between the investigated MPS II families and all the studied MPS II patients except that the patient P2 has a negative family history for MPS II.

Our cohorts were subdivided into two groups: P1-P7 for which we had all the biochemical and molecular data and the other group P8-P12 for which we only had the biochemical data because the patients died before our family surveys to perform a genetic analysis. In our hospital, our approach to studying patients with these rare metabolic diseases is as follows: We systematically perform biochemical analyses and the molecular analysis is performed according to the requests of the couples or families at risk; in this case, written consent will be provided by these families.

Thus, the families gave informed consent before withdrawal of blood and urine samples and written informed consent was obtained and signed by all studied families: For patients less than 16 years old (P1, P5, P6, P7, P8 and P9), the written consent forms were signed by their parents.

In patients older than 16 years (P2, P3, P4, P10, P11 and P12), two subgroups were cited:

In patient who are still alive (P2, P3 and P4), the written consent forms were signed by themselves according the available consent form and in the deceased patients (P10, P11 and P12), the written consent forms were signed by themselves when sampling.

This study was approved by the Ethics Committee of the Fahat Hached Hospital Sousse, Tunisia. All procedures were in accordance with the ethical standards of the responsible committee on human experimentation (institutional and national) and with the Helsinki Declaration of 1975, as revised in 2000 and approved by the Ethics Committees of the respective Tunisian hospitals.

### Biochemical diagnosis

The mother and other female members of each family included in the study were examined in order to create a clearer profile of the disease’s transmission to facilitate prenatal diagnosis and counselling for MPS II in Tunisia.

#### Quantitative analysis of total urinary glycosaminoglycans (GAGs)

The qualitative and quantitative analyses of urinary GAGs were performed according to Chkioua L. et al. [5].

All subjects identified in the “MPS II” risk groups underwent a screening test using a first morning urine sample, which were sent to biochemistry laboratory of Farhat Hached Hospital, for the determination of glycosaminoglycans (GAG).

Urinary GAGs were quantified using a dimethylmethylene blue (DMB) test [6]. The quantity of DMB bound to sulfated glycosaminoglycans was measured via spectrophotometry at wavelength of 656 nm. Electrophoresis on cellulose acetate plate was carried out to identify which type of GAG is present in excess (e.g., dermatan sulfate, heparan sulfate, keratan sulfate). Discontinuous electrophoresis on cellulose acetate plates separated the different GAGs based on their charge and differential solubility in ethanol, and the mucopolysaccharides were visualized by staining with alcian blue [6]. In the case of detection of increased excretion of urinary GAG, an enzymatic assay of a blood sample was enrolled to determine the activity of iduronate-2-sulphatase.

#### Enzyme analysis

Leukocyte IDS activity was performed in Biochemistry Laboratory of Hospital Farhat Hached Sousse, Tunisia and was determined as previously described using the fluorogenic substrate 4-methylumbelliferyl- alpha-iduronide-2-sulfate [7].

### Molecular analysis and DNA sequencing analysis

Peripheral blood was obtained from patients and genomic DNA was isolated using a standard phenol/chloroform procedure [8].

Each of the 9 exons and introns-exons boundaries of the *IDS* gene were systematically amplified and sequenced. However, in patients with family history, only the exons were analysed. Primer sequences and annealing temperature are provided in Table 1.Nevertheless, to avoid the amplification of the *IDS* pseudogene, we have used the primers according which have been previously described [9]. The PCR reactions were performed according to Chkioua et al. [10]. To identify the type and position of the genetic variants, PCR products were purified from excess primers and dNTP with FavorPrep kitTM (Favorgen(R) Biotech Corp) and were sequenced in both forward and reverse directions using the same PCR primers with the Big DyeTerminator v1.1 Cycle Sequencing Kit (Applied Biosystems). The PCR products were purified by Illustra MicroSpin G-50 Columns (GE Healthcare) and electrophoresed on an automated ABI PRISM 310 genetic analyser and interpreted with ChromasPro 2.4.1 software.

**Table 1:** Primers for PCR and DNA sequencing for detection of *IDS* mutations

Primer	Sequence 5′ > 3′	Tm (°C)	Expected products (bp)
F1-IDS	GAGGAGGTCTCTGTGGCTGC	63 .5	376
R1-IDS	AGGGACGGTAGGAAGGAGTG	61 .4	
F2-IDS	CACTCACTATCTCGCTTCCTC	59.8	540
R2-IDS	CCTCTAACAAGATGTCCCG	56.7	
F3-IDS	GGTTACCTAAGAGATGGCAG	57.3	542
R3-IDS	CAGCCTGTGTCCTCCCTAC	61.0	
F4-IDS	GTAGATGAGGAAACTGAGCC	57.3	475
R4-IDS	CTATTCAATGAGTCTGACACG	55.9	
F5-IDS	GCCTGGAAAACAAGAAACACC	57.9	487
R5-IDS	TGGCGATGGCAGGATGTAG	58.8	
F6-IDS	AGGCAGGAGGTGGGGACAG	63.1	607
R6-IDS	CCAGCACTTTGCCTGATAACTC	60.3	
F7-IDS	CTAAGGGGTAGGGATTGGGAG	61.8	440
R7-IDS	ACCCACACCTATCCGTCAAGC	61.8	
F8-IDS	GGTGATGAGTTTCTACTTCCT	55.9	465
R8-IDS	GAGATGTTCAGAAAGCGTG	54.5	
F9-IDS	GTGAGGTGCCGAGGTGGTG	63.1	468
R9-IDS	GGTGCGTATGGAATAGCCC	58.8	
F9–1-IDS	CTTCAGACATCCCTCAGTGG	95.4	283
R9–1-IDS	GCTCTAACTCCTCCTCTCACC	61.8	

## Results

### Clinical features and biochemical analysis

**Fig. 1:**
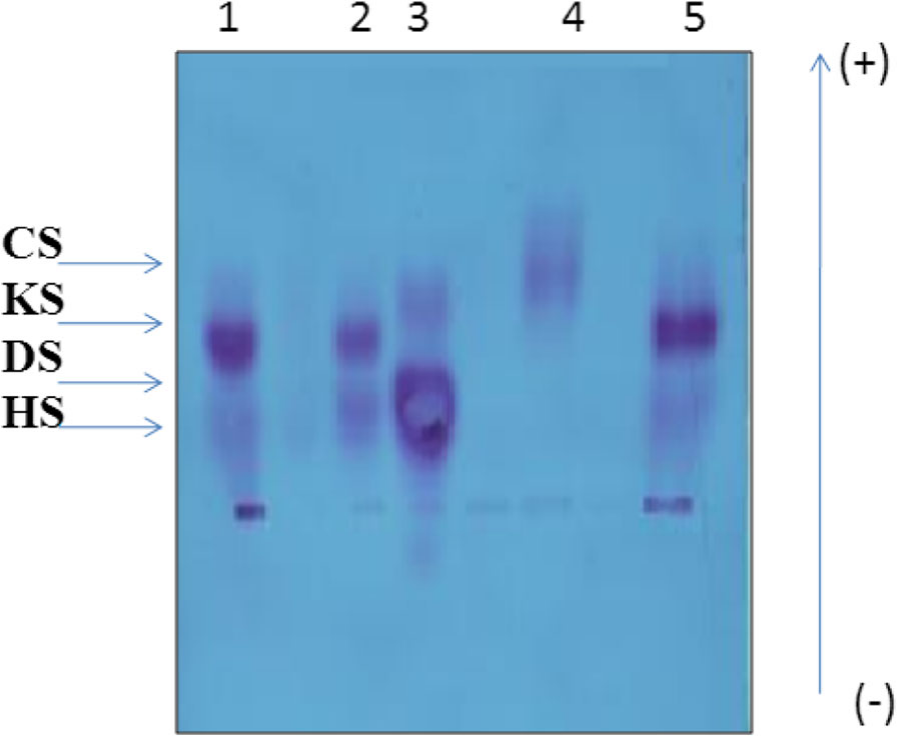
MPS II electrophoresis profile on a cellulose acetate plate of urinary GAGs. 1, 2 and 5: MPS II patients; 3: MPS III patients; 4: Control case; CS: chondroïtin sulphate; DS: dermatansulfate; HS: heparan sulfate, KS: Keratan sulfate

The MPS II patients had a clinical diagnosis of Hunter syndrome that was confirmed by biochemical analyses showing a high concentration of urinary GAGs and deficiency in iduronate 2-sulfatase activity in leukocytes. The electrophoresis profile of urinary GAGs on a cellulose acetate plate is presented in Fig. 1.

Biochemical analysis confirmed the diagnosis of all MPS II patients included in the study. The twelve Tunisian MPS II patients in the present study presented low or undetectable levels ofIDS activity (0.00 to 0.75 nmol/h/mg of leukocyte proteins) (Table 2). The leukocyte enzyme assay for iduronate − 2- sulfatase activity for patients with a severe forme of disease had a mean of 0.16 nmol/h/mg leukocyte protein value, which was < 1% of the enzyme activity of normal individuals (normal mean 2.4 ± 1.1 nmol/h/mg leukocyte protein). The patient with the milder type had an activity of 0.75 nmol/h/mg leukocyte protein.

**Table 2:** Biochemical and molecular MPS II profiles in Tunisian patients

Patients ID	P1	P2	P3	P4	P5	P6	P7	P8	P9	P10	P11	P12
Sex	M	M	M	M	M	M	M	F	M	M	M	M
Consanguinity	unrelated	1st cousins	3rd cousins	unrelated	1st cousins	unrelated	unrelated	1st cousins	unrelated	unrelated	unrelated	1st cousins
Origin	Tunis	Sfax	Kairouan	Sousse	Tunis	Sfax	Beja	Monastir	Sousse	Sousse	Mahdia	Sousse
Urine GAGs^1^ mg/g/creatinine	93.4	30.0	95.0	48.0	56.8	105	116	125	28.4	65.8	23.3	83.9
Age at diagnosis (years/months)	4	1 /6	4	6	3	4/ 2	4	3	2	3	9	12
Actual age of patients (years)	4	18	22	26	5	5	7	9 (died)	9 (died)	19 (died)	29 (died)	39 (died)
Leukocytes IDS activity (nmol/h/mg protein)	0.20	0.20	0.50	0.00	0.750	0.00	0.00	0.39	0.059	0.00	0.65	1.5
Mutations	c.240 + 1 G > A	p.R88P	Ex1_7del^a^	p.Q396*	p.G94D	p.D450Nfs*95	p.Q204*	p.R88P^b^	None found	None found	None found	None found
Location	INTRON 2	EXON 3	*	EXON 9	EXON 3	EXON9	EXON 5	EXON 3				
Restriction enzyme	(−) ECO64I	(−) ACC II	–	(−) Cac8I	(−) BseNI	(−) BamHI	(−) Cac8I	(−) ACC II	These patients were dead before our molecular analysis.			
Fragment length (bp)	N: 373 M: 180 + 193	N: 91 + 432 M: 523	–	N: 49 + 54 + 119 + 333 M: 95 + 119 + 173	N: 37 + 46 + 109 + 331 M: 37 + 155 + 331	N: 137 + 303 M: 440	N: 200 + 280	N: 91 + 432 M: 523				
Status	reported	reported	reported	reported	reported	Novel	Novel	reported				
Reference	[11]	[12]	[10]	[11]	[4]	This report	This report	[12]				
Polymorphisms		c.419–16 delT; c.641C > T (p.T214M); c.438 C > T (p.T146T); c.709-87G > A; c.1006 + 38 T > C	none	none	c.419–16 delT; c.641C > T (p.T214M); c.438 C > T (p.T146T); c.709-87G > A; c.1006 + 38 T > C	c.419–16 delT; c.641C > T (p.T214M); c.438 C > T (p.T146T); c.709-87G > A; c.1006 + 38 T > C	c.419–16 delT; c.641C > T (p.T214M); c.438 C > T (p.T146T); c.709-87G > A; c.1006 + 38 T > C					
Phenotype	severe	severe	Severe	severe	Mild	severe	Severe	severe				

1Urine GAGs: normal value GAGs

^a^: at position 1,307,880 (GenBank NT:019686), and the distal deletion breakpoint was located at position 1,346,697

*N* Normal sequence; *M* Mutated sequence.

^b^: According to his phenotype, it can be presumed that the P8 was homozygous for p.R88P mutation.

* indicates the stop codon according the standard nomenclature of the nonsense mutation

Clinical characteristics and identified genotypes are summarized in Tables 2 and 3. On physical examination, all patients were found to have hepatosplenomegaly, coarse facies and cranial dysmorphism. Thus, psychomotor retardation and dysostosis multiplex were severe in patients P2, P3, P7, P9 and P10. Mental retardation and cardio-respiratory involvement were observed in all patients; nevertheless these clinical features were more severe in patient P7 which had higher GAG concentration, according to the age at diagnosis. The level of GAG in patients with milder form of disease was lower than in the severe form.

**Table 3:** Clinical findings of the MPS II patients

MPS II patients	P1	P2	P3	P4	P5	P6	P7	P8	P9	P10	P11	P12
Age at diagnosis (year/month)	4	1/6	4	6	3	4/2	4	3	2	3	9	12
Age (years)	4	18	22	26	5	5	7	9 (died)	9 (died)	19 (died)	29 (died)	39 (died)
**Recurrent clinical symptoms**												
**Hepatosplenomegaly**	++	++	++	++	+	++	++	++	++	++		++
**Coarse facies**: **Broad noses, Macroglossia**	++	++	++	++	++	++	++	++	++	++	++	++
**Cranial dysmorphism**: **macrocrania**	++	+++	+++	++	++	++	++	+	++	++	++	++
**Psychomotor retardation**	++	+++	+++	++	+	++	+++	++	+++	+++	++	++
multiple dysostosis**: joint stiffness, oval vertebrae**	++	+++	+++	++	++	++	+++	+++	+++	+++	+++	+++
**Osteopenia**	++	++	++	+++	++	+++	++	++	++	++	+++	
**Mental retardation**	++	+++	++	++	+	++	++++	++				++
**Respiratory problems: nasal obstruction, sleep apnea**	++	++	++	++	++	++++	++++	++	++	++	++	++
**Causes aggravating respiratory problems: otitis, large language and adenoids.**	++	++	++	++	++	++++	++++	++	++	++	++	++
**Cardiovascular involvement: arrhythmia and congestive heart failure**	++	++	++	++	++	+	++++	++	++	++	++	++
**Specific clinical symptoms**												
	Multiple hernia			Blood smear shows an overload	Blood smear shows an overload Dysphasia		Skin involvement					Rhinorrhea umbilical hernia

### *IDS* mutation analysis

We analysed the *IDS* gene of twelve MPS II patients from different regions of Tunisia using PCR, RFLP-PCR, and direct sequencing methods. Clinical and identified genotypes are summarized in Tables 2 and 3.

**Fig. 2:**
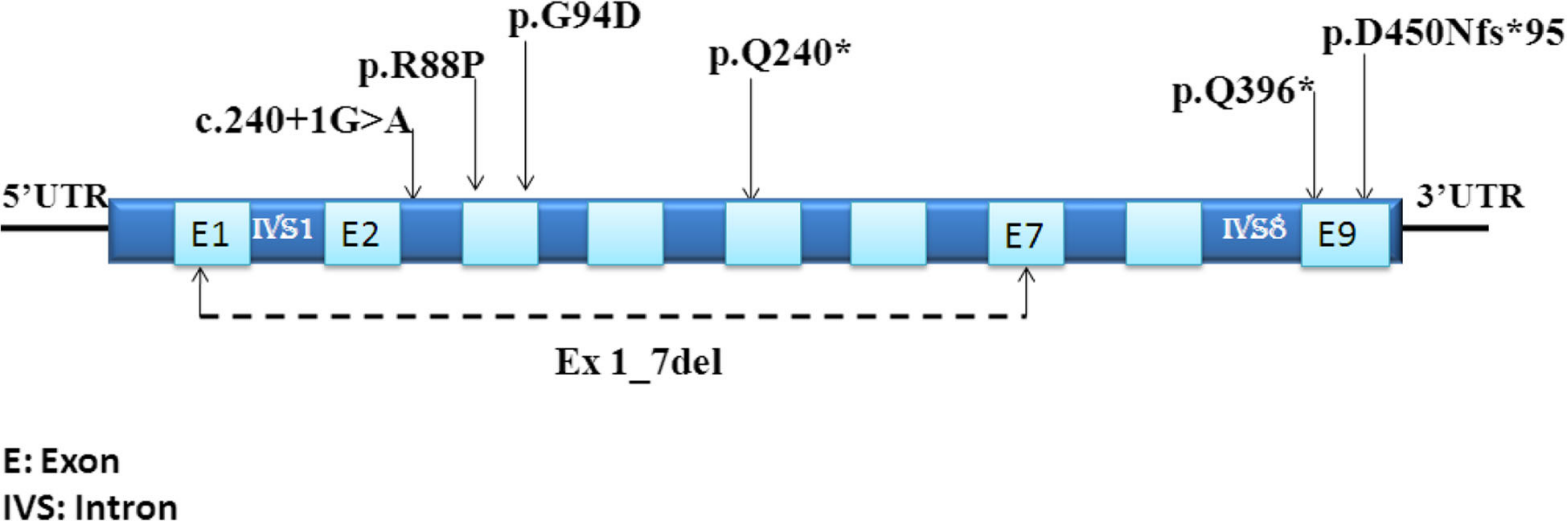
Positions of *IDS* mutations

From the DNA sequencing analysis and RFLP-PCR, two novel mutations and five previously reported mutations were identified (Fig. 2). These included two missense mutations p.R88P (−) AccII and p.G940D (+) Cac8I, one splice site mutation c.240 + 1G > A (−) Eco64I, one nonsense mutation p.Q396*, and one large deletion skipping the exon 1 to 7 of *IDS* gene, ex1_7del at position 1,307,880 (GenBank NT: 019686); the distal deletion breakpoint was located at position 1,346,697 [13]. The two unreported mutations were p.D450Nfs*95 (−) BamHI and p.Q204* (−) cac8I (Table 3) and were probably pathogenic according to the bioinformatic tools such as Entprise *web-server, (**http://cssb2.biology.gatech.edu/ENTPRISE/**).*

To confirm that these alterations are new mutations we have analyzed 50 genomic DNA by sequencing and all showed the normal allele.

The novel frameshift mutation in exon 9 is caused by a single-base deletion of guanine at genomic DNA position 1565 and changed codon 450 for aspartic acid (GAT) to a chain termination resulting, in premature termination codon (TAG) of the enzyme glycoprotein. The novel nonsense mutation p.Q204* in exon 5 was due to a substitution of cytosine by thymine at position 610 of the cDNA and changed codon 240 for glutamic acid (CAA) to a premature termination codon (TAA).

The screening of polymorphisms in *IDS* gene revealed that there are at least 5 polymorphisms detected: c.419–16 delT, c.641C > T (p.T214M), c.438 C > T (p.T146T), c.709-87G > A, and c.1006 + 38 T > C. Here, we have demonstrated that the same polymorphisms were associated with severe (P2 and P4) and or mild (P5) phenotype.

## Discussion

### Clinical, biochemical and molecular correlations in MPS II patients

All of the data was available for the MPS II patients except for patients P9-P12 for whom only clinical and biochemical data were known because these patients died before their molecular data could be collected. The delay in diagnosis was explained by a lack of awareness among physicians of the specific MPS II clinical features associated with the adverse socioeconomic conditions of those patients.

Based on the clinical, biochemical, and molecular data, 6 patients (P1-P4 and P6–7) were classified in the MPS II group with a severe disease, and only one patient (P5) presented a mild phenotype.

According to the clinical data, the confirmation of diagnosis in all MPS II patients was done at a mean age of 5 years, unlike what is found in the literature [1].

The urinary GAG concentration ranged from 30.0 to 125 mg of creatinine, according to the age of each patient. The high level of heparan sulfate in the urine was correlated with the severity of the disease as previously described by Tomastsu S et al., who demonstrated a significant correlation between the level of heparan sulfate and the severity of this disease [14].

The leukocyte IDS activity in patients (P1-P4; P6-P8) with the severe type of the disease had a mean of 0.13 nmol/h/mg of proteins. Based on the high level of urinary GAGs and the deficiency of IDS activity, a relationship seems to exist between these data and the phenotypic expression of Hunter syndrome, contrasting with what is reported in the literature such as in Filipino patients [14]. However, the clinical profiles of the MPS II patients (P1-P7) were in agreement with several studies described in the literature, and the clinical manifestations of the phenotype of Hunter syndrome ranged from moderate to severe Hunter syndrome phenotypes [15].

The most recurrent symptoms observed in this series ranged in degree of severity, including hepatosplenomegaly, coarse facial features including broad noses, macroglossia, psychomotor and mental retardation, multiple dysostoses including joint stiffness, oval vertebrae, respiratory problems including otitis, nasal obstruction, and enlarged tongue and adenoids.

Patient P8 has a female gender related to Patient P2 who was hemizygous for the p.R88P mutation. She presented GAG excretion of 125 mg/g/creatinine and leukocyte IDS activity of 1.00%. She died before molecular analysis was conducted, but she probably had the same genetic mutation as patient P2 since he presented the same clinical profile as her cousin P8. The existence of intrafamilial phenotypic heterogeneity suggests that the presence of genetic and epigenetic polymorphisms could be important to confirm their effects on the phenotypic expression of the disease. MPS II females have been noted to present very rare clinical descriptions, and most of them present the severe form of the disease [16]. Importantly, the identification of MPS II heterozygous females by measurement of IDS activity and urinary GAG levels is unreliable. Therefore, the definitive diagnosis should be determined using genetic analysis [17].

Previous studies [18, 19] showed that the phenotypic expression of this disease in MPS II females is uncommon, and most of the cases described in the literature presented the severe phenotype. MPS II heterozygous females are rarely reported except for the presence of double mutant alleles or a coincidental genetic defect, leading to skewed X-inactivation or hemizygosity in heterozygotes [20].

Patient P12 was diagnosed at the age of 3 years old when he had an inguinal hernia operation. However, coarse facial features, including macrocrania, macroglossia and small teeth, had been noted at the age of 18 months. He presented severe hepatosplenomegaly, skeletal disease, and severe mental retardation. The biochemical test showed that the leukocyte IDS activity in this patient was significantly higher (1.5 nmol/h/mg of proteins) than the enzyme activity of other MPS II patients. Patient P12 presented the severe phenotype of the MPS II disease, but he died before the molecular analysis hence the interest of carrier testing.

In this study, cardiovascular involvement, including arrhythmia and congestive heart failure, was identified in all MPS II patients and has been shown to be the cause of morbidity and mortality in most patients, as has been described previously in the literature [21].

Seven different mutations were found in the 12 MPS II patients. These nucleotides variations reflect the genetic heterogeneity leading to the wide spectrum of clinical phenotypes of MPS II in agreement with several other studies [4, 15].

Sequence alterations in the *IDS* gene included five previously reported mutations and two novel mutations. The severe phenotype was found in patients who had the following mutations: c.240 + 1G > A, p.R88P, Ex1_7del, and p.Q396*. This in agreement with several previous studies (Table 3).

The missense mutation p.G94D was associated with a milder phenotype. This finding agrees with the data reported in Australian patients [4, 22]. This mutation occurred within a conserved amino acid of human lysosomal sulfatase, which is essential for the common sulfatase activity [23].

The p.R88P and p.G94D missense mutations, associated with the same polymorphisms were identified in two patients P2 and P5 who presented two different phenotypes. The genotype heterogeneity in MPS II could be explained by the possible contribution of other genes in the severity of the phenotype in patient P2.

The first novel alteration c.610C > (T p.Q204*) was a nonsense mutation and was identified in a patient who developed a severe form of MPS II. This mutation was due to a cytosine -to- thymine transversion at position 610 of the cDNA resulting in premature glycopolypeptide truncation at the 204th codon in exon 5 of *IDS* gene. Carrier testing was performed in the mother, who was found conductive.

The second novel frame shift mutation (p.D450Nfs*95) in exon 9 of the *IDS* gene is caused by a single-base deletion of guanine at genomic DNA position 1565. This mutation in exon 9 changes codon 450 from aspartic acid (GAT) to a chain termination codon (TAG) that leads to the lack of 95 amino acids at the amino terminus of the IDS protein. This novel mutation may lead misfolding of the glycopeptide resulting in a non-functional protein.

Mutations leading to a premature translation codon have frequently been classified as severe mutations; in agreement with this, the novel frame shift (p.D450Nfs*95) mutation was found in a patient (P6) who presented the severe phenotype.

To probe effects of various amino acid substitutions on catalytic activity or stability of IDS protein, we located the mutations (p.Q240* and p.D450Nfs*95) on the *IDS* model and characterized their structural effects.

**Fig. 3:**
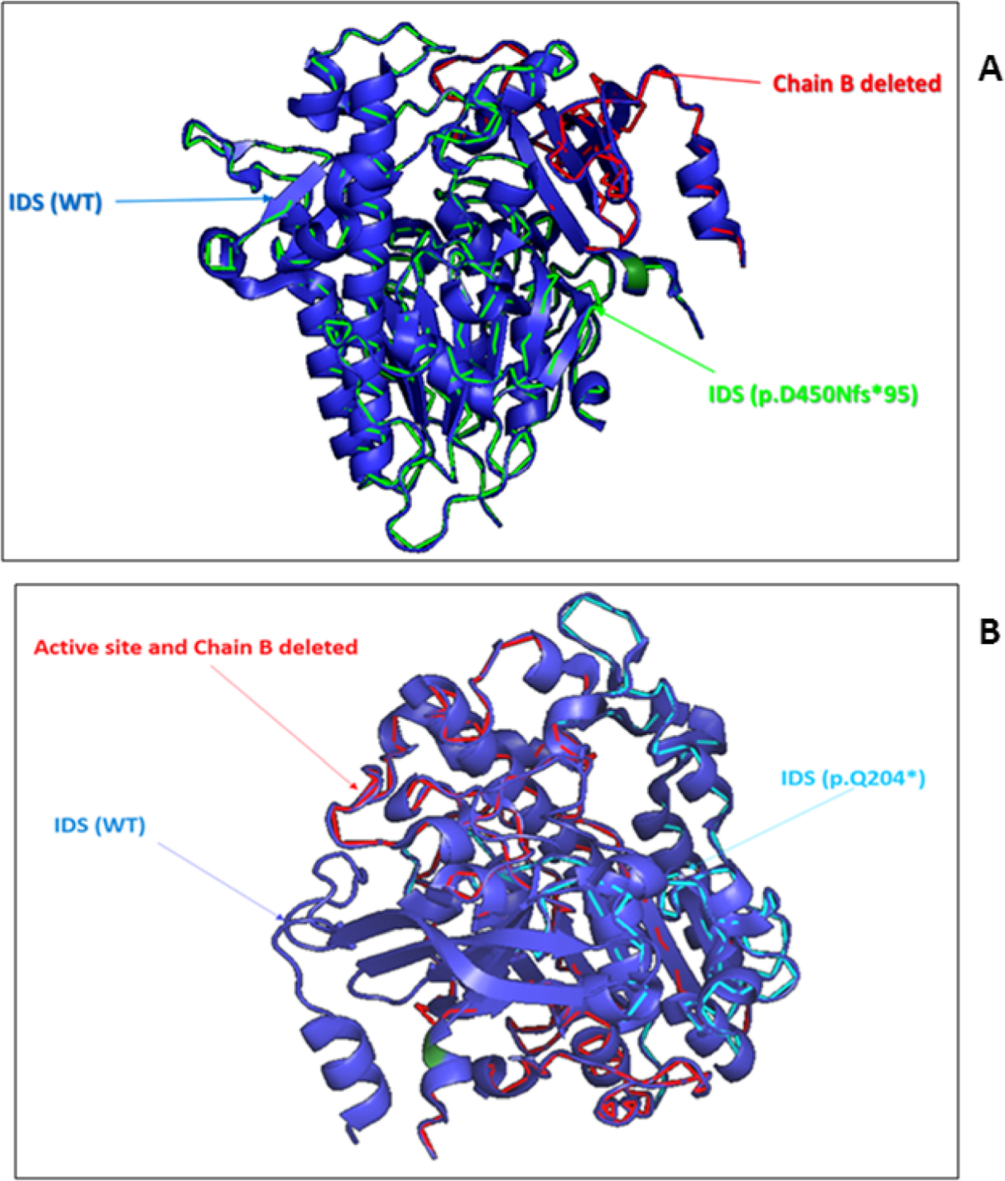
**a**: Structural superposition of wild type (blue) and the p.D450Nfs*95 mutated IDS protein (green). The deleted part of the mutate protein (red) correspond the C-terminal of the heavy chain (5 amino acids) and the all part of the light chain (14KDa). PyMOL; pdb = 5FQL. **b** Structural superposition of wild type (blue) and the p.Q204* mutated IDS protein (light blue). The deleted part of the mutate protein (red) correspond the active site and the C-terminal of the heavy chain PyMOL; pdb = 5FQL

The p.D450Nfs*95 mutation results in exon skipping and introducing premature translation termination codon in exon nine with an abnormal IDS protein and have been classified as severe mutation. The premature stop codon causes a deletion of the last 5 amino acids of the heavy chain which contains the catalytic core (451□455) and the entire light chain (456□550) of IDS protein (Fig. 3a). The predicted premature stop codon could affects protein stability (www.pymol.org - PyMOL; PyMol (pdb: 5FQL)).In fact, the light chain of the IDS protein had an important role in the stability of the protein. Furthermore, the four antiparallel strands comprising the light chain are considerably longer than those of other sulfatases, and hence a greater contribution to the shape of the substrate-binding cleft comes directly from the light chain [24]. The expected severity of this mutation was variable and consequence range from local destabilization and misfolding to global unfolding, leading to premature degradation. The K479 residue in the exon 9 was important to the substrate binding [24]. The lack of this residue in our patient (P6) with p.D450Nfs*95 mutation result the non-functional IDS protein by the absence substrate binding. Moreover, three frame shift mutations were described in the exon 9 of *IDS* gene: p.R443X, p.R443X, p.Y466X and found in the patients who presented severe phenotype [25, 26]. However, investigation of mRNA and expression studies will be necessary to prove this conclusively. Correlation between genotype and phenotype was uncertain using genomic DNA. Further investigations such as transcription tests are useful to predict with confidence the disease phenotype.

Several described mutations directly affect the catalytic core of the enzyme, for example by direct substitution of key active-site residues [27]. In this study, the nonsense mutation p.Q204* was located on the light chain of the IDS protein, downstream of the catalytic core which contains 451–455 residues, destroys the enzyme activity and the β chain of the IDS protein (www.pymol.org - PyMOL; PyMol (pdb: 5FQL)) (Fig. 3b). Consistent with this concept, this mutation was associated with the severe form of MPS II observed in patient P7.

In this study, there was no relationship between the genotype and phenotype in these MPS II patients except for the significant correlation between the high level of urine GAGs and the severity of the disease. Further studies including a large number of cases in Tunisian population of the same age and genotype are needed in the first time, to confirm this correlation in MPS II patients and in the second time to screening of haplotyping data for the recurrent mutation because of a founder effect.

## Conclusion

In conclusion, this paper provides additional information on the clinical, biochemical and molecular correlations in MPS II patients. Multidisciplinary approaches, such as carrier detection and genetic counselling, are needed for the parents that do not know their *IDS* genetic profile in order to decrease the prevalence of this inherited pathology and also to prevent the early death of patients.

## Data Availability

The datasets used and analyzed during the current study are available from the corresponding author upon request. The mutations of the MPSII patients were submitted to ClinVar database (https://www.ncbi.nlm.nih.gov/clinvar/) under accession number SCV001194274 and SCV001194273.

## Abbreviations

MPS II: Mucopolysaccharidosis type II
*IDS*: Iduronate 2-sulfatase
GAGs: Glycosaminoglycans
HS: Heparane sulfate
DS: Dermatan sulfate
